# Desmoplastic fibroma of the mandible - review of the literature and presentation of a rare case

**DOI:** 10.1186/1746-160X-5-25

**Published:** 2009-11-24

**Authors:** Michael Schneider, André C Zimmermann, Rita A Depprich, Norbert R Kübler, Rainer Engers, Christian D Naujoks, Jörg Handschel

**Affiliations:** 1 Department for Cranio- and Maxillofacial Surgery, Heinrich-Heine-University, Moorenstr. 5, D-40225 Düsseldorf, Germany; 2 Department of Pathology, Heinrich-Heine-University, Moorenstr. 5, D-40225 Düsseldorf, Germany

## Abstract

Desmoplastic fibroma (DF) is a rare, benign but locally aggressive, intraosseous lesion with a high tendency of local recurrence. In this report the actual literature is reviewed regarding epidemiological data, pathology, clinical diagnostic criterias, therapy and prognosis. Moreover, a report of an interesting case is included localized in the mandibular corpus.

## Introduction

Desmoplastic fibroma (DF) is a benign but locally aggressive neoplasm of the bones [1,2] and it is very rare in the mandible like some other intraoral tumours [3]. There is no metastasis but beside of their destructive growth they show a high frequent recurrence after local resection [4]. In 1838 the German physiologist and anatomist Johannes Müller [5] characterised the term „desmoid“ (Greek: „desmos“ = „band/ligament“). In 1958 Jaffe [6] firstly described a primarily osseous-arised aggressive fibromatosis of the femur, the tibia and also of the scapula. He declared these tumours as „desmoplastic fibromas“. In 1965 the first report about a desmoplastic fibroma of the jaw was presented by Griffith und Irby [7] and since that time a considerable number of similar cases have been published [2,4,8-40]. In jaw area non-odontogenetic fibromatosis was declared as desmoplastic fibroma what distinguished it from odontogenic fibroma [1,36,41,42].

For reviewing the international literature a systematic search in the PupMed database of the National Library of Medicine was performed using the key words "desmoplastic fibroma", "mandible" and "jaw". This search revealed that only 76 cases (from 1968 to 2009) of desmoplastic fibroma in the jaw area have been published to date, which reconfirms the infrequency of these tumours and the low incidence.

Intraosseous desmoplastic fibromas (DF) are very rare myofibroblastic tumours (far less than 1% of all bone tumours) [43,44] and they can occur in every bone of the body. In 22% of all cases the mandible is mostly affected [4,43]. These benign but locally agressive lesions offer many similarities to soft-tissue DF [41]. The incidence of desmoplastic fibroma of the jaw is equal in male and female patients [45]. On average, patients are 15.1 years old at the time of the final diagnosis [46]. Pathognomonic symptoms do not exist and their occurence is mostly insidious. Some cases described pain and swellings [47-49]. Radiologic findings are unspecific and extend from mono- to polycystic appearance with a partially sharp or diffuse borderline [50]. Magnetic resonance imaging can clearly distinguish between intraosseous tumours and normal bone marrow and is particularly suitable for surgical planning [43]. As therapy, surgical resections, radiotherapy and if necessary, pharmacological treatments are recommended. In respect of the high recurrence rate, surgical resection is the most favourite option [44,51,52], but depending on tumour localisation (e.g. cerebric) or resulting mutilations it is not always feasible. In cases of non-in-sano resected fibromatosis the recurrence rate can be lowered significantly by adjuvant radiotherapy [53]. However, the prospected mutagenic effects makeradiotherapy not suitable as a solitary treatment [47]

In this report we present the clinical course and therapy of a patient with the first diagnosis of a desmoplastic fibroma in the left mandibular corpus, after resection of an extensive but low-grade myofibroblastic sarcoma in the right ramus 8 years before. Regarding the mental nerve we decided on a gentle resection and an observing follow-up strategy after the final diagnosis had been confirmed. Ten months after resection, clinical and radiographical controls of our patient showed no recurrence of the DF, but a periodic follow-up over at least 3 years is recommended [54]

## Case Report

**Figure 1:**
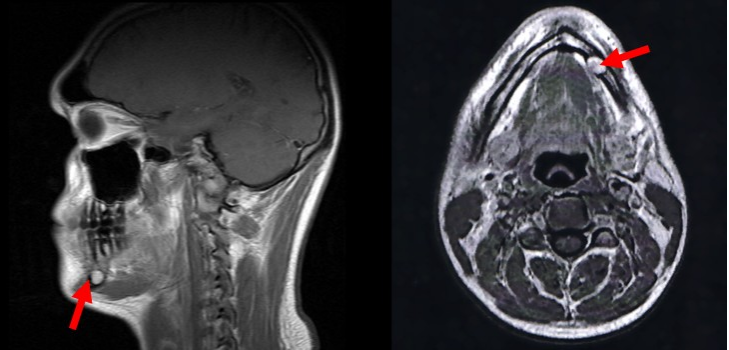
**MRI (T1-weighted) illustrating a 10 mm tumour within the left mandibular corpus (red arrow)**.

**Figure 2:**
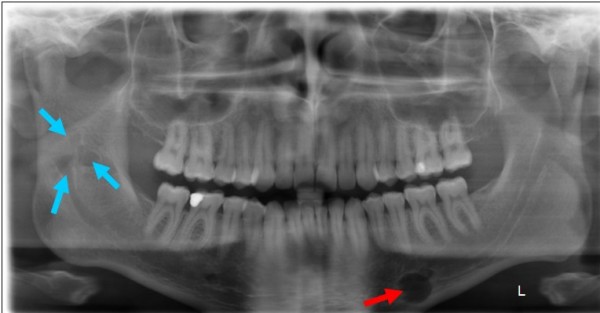
**Panoramic radiography (patient, 23 years): 13 mm osteolysis in left mandible (red arrow)**. Noted translucency within the right ramus (blue arrow).

A 23-year-old Caucasian male patient consulted the Department for Cranio- and Maxillofacial Surgery for analysing an intraosseus, rounded tumour in the left mandibular corpus, which was initially diagnosed by an MRI scan 12 months ago in a different institute (Fig. 1). This MRI was part of a routinely follow-up, since 10 years before two intracranial, solid and non-proliferating soft-tissue-tumours were diagnosed in the right cerebellar hemisphere (close to the foramen magnum) and underneath the left temporal lobe. Additionally, two years later an extensive but low-grade myofibroblastic sarcoma in the right ramus of the mandible was resected. A current panoramic radiography (Fig. 2) showed a well circumscribed, rounded osteolysis with a diameter of 13 mm in immediate proximity to the left mental foramen. Besides, the known and in size constant translucency within the right ramus (after sarcoma-resection 8 years before) appeared inconspicuously. There were no other pathological findings.

**Figure 3:**
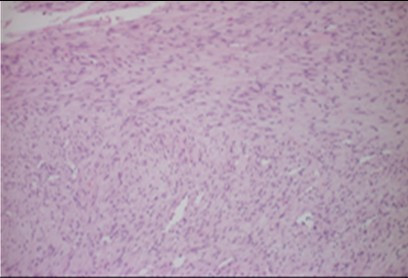
**Partially parallel or plexiform arranged spindelcells with slim and elongated nucleus without cytological sings of malignity (HE-staining; original magnification: 100×)**.

**Figure 4:**
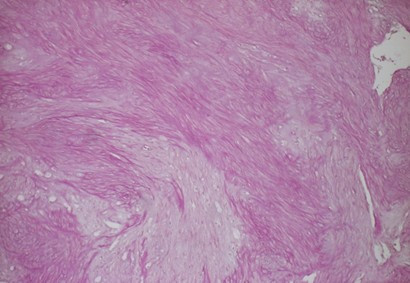
**High-grade formation of collagen fibres (red) (EvG-staining; original magnification: 100×)**.

**Figure 5:**
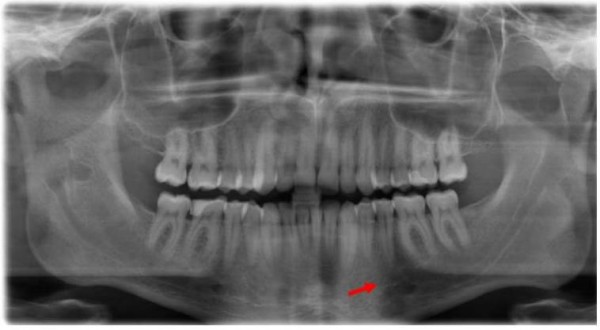
**Panoramic radiography (10 month after resection): No recurrence and obvious ossification in the area of the former osteolysis (red arrow)**.

As therapy a paramarginal approach showed the intact vestibular cortical surface with an inconspicuous mental foramen and a normal structured mental nerve. The osteotomy exposed a rounded cavity, in which a 1.3 × 0.9 × 0.6 cm sized, greying, tubercular, firm-elastic conjunctive-tissue-tumour was located. This tumour showed a very slight adherence to the surrounding bone and was resected easily. After that the bone cavity was carefully reamed under preservation of the mental nerve. Because of the small defect-size any bone-grafting material could be dispensed. The histopathological examination revealed a mesenchymal tumor, composed of spindle-shaped cells with myofibroblastic differentiation, abundant collagen formation and low proliferation activity (Fig. 3 and 4). In immunohistochemical stainings the tumour-cells showed a positive reaction for smooth muscle actin and a negative reaction for S100 (data not shown). With the proliferation marker Mib1 less than 5% of the tumour-cells proved to be positive. Based on these characteristics the diagnosis of a desmoplastic fibroma was made, and this diagnosis was confirmed by a reference institute. The post-operative recovery was normal based on clinical examination The radiographical (panoramic X-ray) follow-up showed an obvious ossification of the former resection cavity (Fig. 5) and the patient described no hypaesthesia of the mental nerve at any time.

In conclusion the Desmoplastic fibroma (DF) is a rare, benign but locally aggressive, intraosseous lesion with a high tendency of local recurrence. With respect of the patient's post-operative well-being and if periodic follow-ups are guaranteed, the tumor should be carefully resected with only narrow safety margins.

## Competing interests

All authors disclaim any financial or non-financial interests or commercial associations that might pose or create a conflict of interest with information presented in this manuscript.

## Authors' contributions

MS, AZ, RD, CN and JH made substantial contribution to the conception and design of the manuscript. RE carried out the pathological investigations and participated in creating this part of the manuscript.

All authors were involved in revising the manuscript critically and have given final approval of the version to be published.

## Consent

Written informed consent was obtained from the patient for publication of this case report and accompanying images. A copy of the written consent is available for review by the Editor-in-Chief of this journal
